# Cardioepigenetics in action: aerobic exercise-induced modulation of miRNAs, lncRNAs, and chromatin remodeling in cardiovascular disease

**DOI:** 10.3389/fcvm.2025.1579352

**Published:** 2025-08-01

**Authors:** Shoudu Yuan, Qi Ye, Ran Qin

**Affiliations:** ^1^ School of Physical Education and Health, Chongqing College of International Business and Economics, Chongqing, China; ^2^ Physical Education Institute, Chongqing College of Humanities, Science & Technology, Chongqing, China; ^3^ Southwest University Hospital, Southwest University, Chongqing, China

**Keywords:** microRNAs, long non-coding RNAs, epigenetic modifications, cardioprotection, exercise

## Abstract

Cardiovascular diseases (CVDs) remain a leading cause of morbidity and mortality worldwide, despite advances in prevention and therapy. Emerging evidence highlights the central role of epigenetic modifications and non-coding RNAs including microRNAs (miRNAs) and long non-coding RNAs (lncRNAs) in the regulation of gene expression networks underlying cardiovascular homeostasis and disease. Concurrently, physical exercise has been recognized not only as a preventive and therapeutic strategy for CVDs but also as a potent modulator of epigenetic landscapes. This review explores the mechanistic links between aerobic exercise and epigenetic modulation, focusing on how structured physical activity influences the expression and function of miRNAs and lncRNAs, as well as chromatin remodeling processes in cardiovascular tissues. We provide a comprehensive overview of aerobic exercise-responsive non-coding RNAs implicated in vascular inflammation, endothelial function, cardiac remodeling, myocardial infarction, and atherosclerosis. Additionally, we discuss aerobic exercise-induced changes in DNA methylation and histone modification patterns that contribute to transcriptional reprogramming and long-term cardiovascular benefits. Finally, the review evaluates the translational potential of targeting aerobic exercise-regulated epigenetic factors for early diagnosis, risk stratification, and personalized therapies in CVD management. Understanding the molecular underpinnings of cardioepigenetic responses to exercise opens promising avenues for precision cardiovascular medicine and integrative therapeutic strategies.

## 1 Introduction

Cardiovascular diseases (CVDs) are still the most important causes of global morbidity and mortality, responsible for a high proportion of healthcare problems and afflicting millions of people every year (1). Increasing incidence of CVDs is closely linked with a range of risk factors, including both modifiable lifestyle-related behaviors (such as physical inactivity, poor diet, smoking, and alcohol consumption) and intermediate clinical conditions (such as obesity, type 2 diabetes, and hypertension) that are influenced by those behaviors (2, 3). In the wake of the emerging public health crisis, non-pharmacological interventions, and especially habitual physical exercise, particularly aerobic training, have garnered significant attention due to their profound cardioprotective effects (4). Research has reported that exercise exerts a positive impact on cardiovascular health via multifaceted mechanisms, such as the enhancement in cardiac function, the reduction in systemic inflammation, and the minimization of oxidative stress (5). Although the physiological effects of exercise are well documented, the molecular foundations of its cardioprotective effects are a subject of keen research. Recent studies have progressively concentrated on the function of non-coding RNAs (ncRNAs), such as microRNAs (miRNAs) and long non-coding RNAs (lncRNAs), as essential regulators of gene expression within the setting of cardiovascular adaptation to exercise (6). Such ncRNAs are presently understood to be key mediators of cellular mechanisms like angiogenesis, apoptosis, fibrosis, and hypertrophy, which are all inherent components of cardiovascular homeostasis (7, 8). For instance, some miRNAs, such as miR-1, miR-133, and miR-206, have been shown to modulate cardiac remodeling and function in response to exercise. Similarly, lncRNAs, through mechanisms involving interaction with chromatin, proteins, and other RNAs, have been shown to modulate gene expression networks that impact cardiovascular health (9). The modulation of these non-coding RNAs following physical exercise highlights their value as biomarkers and therapeutic targets for cardiovascular disease prevention and therapy.

Besides ncRNAs, other major epigenetic modifications include DNA methylation and histone modification have emerged as another leading way through which exercise exerts its cardioprotective effects (10–12). It has been demonstrated that exercise modifies the DNA methylation pattern, especially genes implicated in inflammation, oxidative stress, and metabolic regulation (13–15). Analogously, exercise-promoted changes in histone acetylation and methylation have been associated with the activation of cardioprotective gene programs (16). These epigenetic modifications provide not only a mechanistic basis for the long-term benefits of exercise but also point to a possibility of using exercise as a means to modulate gene expression in a heritable yet reversible manner (17). The present review aims to update the knowledge of the cardioprotective role of physical exercise, focusing particular attention on miRNAs, lncRNAs, and epigenetic modifications contributing to this function. In this review, we focus on the molecular pathways underlying cardiovascular adaptations induced by aerobic exercise by integrating the latest findings in this rapidly evolving field. We highlight the role of non-coding RNAs (miRNAs and lncRNAs) and epigenetic modifications as key mediators of these adaptations, emphasizing their potential as biomarkers and therapeutic targets. A deeper understanding of how aerobic exercise modulates these molecular mechanisms may pave the way for innovative strategies in the prevention and management of CVD, addressing one of the foremost global health challenges.

## 2 miRNAs and cardioprotection *via* exercise

### 2.1 Overview of miRNAs

miRNAs are small, non-coding RNA molecules that control gene expression after transcription by binding to target messenger RNAs (mRNAs) to suppress their translation or promote degradation (8). In the cardiovascular system, miRNAs play crucial roles in various processes, from cardiac development, hypertrophy, fibrosis, and angiogenesis. Dysregulation of specific miRNAs has been implicated in the pathogenesis of cardiovascular disease, including heart failure, myocardial infarction (MI), and atherosclerosis (18). With the potential to modulate multiple pathways simultaneously, miRNAs have been identified as key regulators of cardiac homeostasis (19). Exercise has been shown to influence miRNA expression patterns, suggesting that these molecules contribute to the molecular adaptations that underlie exercise-induced cardioprotection (20).

### 2.2 Exercise-induced miRNA changes in cardiovascular health

Exercise training has been demonstrated to induce profound and active alterations in the expression levels of miRNAs that are crucial for the regulation of heart function and assisting the body in coping with stress (21). Exercise-induced alteration in miRNA expression is implicated in important biological processes, including cardiac remodeling, inflammation, oxidative stress, and endothelial function, all of which are critical for maintaining cardiovascular health (22). A number of studies have found that certain miRNAs increase or decrease in response to exercise, showing their involvement in the cardiovascular system's adaptation. For example, aerobic exercise upregulates cardiac miR-126 expression, an established miRNA found in endothelial cells (23), as demonstrated in streptozotocin-induced diabetic male Wistar rats (8–10 weeks old) subjected to treadmill running (60 min/day, 5 days/week for 8 weeks), resulting in enhanced vascular endothelial growth factor (VEGF) signaling and angiogenesis in cardiac tissue (24). This illustrates how important miR-126 is in allowing exercise to improve the health of the heart, specifically by affecting the creation of new blood vessels and vascular repair mechanisms.

Analogously, Fathi et al. showed that 14 weeks of endurance training (treadmill running at ∼75% VO_2_max) in male rats led to physiological cardiac hypertrophy, along with a significant upregulation of miR-1 and miR-133. These changes were associated with improved cardiac function and regulation of apoptosis- and growth-related genes, suggesting a role for these miRNAs in exercise-induced cardiac adaptation (25). Additionally, miR-21, implicated in cardiac hypertrophy and fibrosis, has been modulated by an acute exhaustive exercise in ways that reduce pathological remodeling and enhance survival of cardiomyocytes in chronic heart failure patients (26). These miRNA alterations collectively enhance cardiovascular adaptation by promoting cardiac function, supporting vascular regeneration, and reducing the risk of MI and heart failure.

The mechanisms underlying exercise-induced changes in miRNA expression are complex and involve multiple levels of regulation, including modulation of the miRNA biogenesis machinery. Key proteins in this process Drosha, Dicer, and Argonaute (AGO) orchestrate the sequential processing of primary miRNA transcripts (pri-miRNAs) into mature, functional miRNAs (20). Endurance training has been shown to upregulate Dicer and AGO2 expression in metabolically active tissues such as adipose and skeletal muscle, primarily via activation of AMP-activated protein kinase (AMPK), a central energy sensor responsive to increased energy demand during exercise (27). Concurrently, reactive oxygen species (ROS) generated during aerobic activity play a dual regulatory role; at moderate levels, they enhance the expression of Drosha, Dicer, and Exportin-5, promoting efficient miRNA processing, whereas excessive ROS may impair Dicer and AGO2 stability (28). These effects facilitate the maturation of cardioprotective miRNAs such as miR-1 and miR-133a, which contribute to improved cardiac function and stress adaptation in response to endurance exercise.

These molecular changes are further orchestrated by several upstream signaling pathways that integrate physiological stimuli such as mechanical stress, metabolic shifts, and redox changes. For instance, PGC-1α pathway, activated by aerobic training, co-regulates miRNA programs; notably, it modulates miR-696, which targets PGC-1α and influences muscle glucose metabolism (29). Given that NF-κB is a known transcriptional activator of miR-146a and miR-155 in inflammatory contexts, and that these miRNAs increase following endurance exercise, it is plausible that NF-κB contributes to their upregulation during exercise-induced immune modulation (30, 31).

Wu (32) collectively, these signaling cascades including PGC-1α and NF-κB integrate mechanical, metabolic, and oxidative stimuli to finely regulate miRNA biogenesis and expression in a tissue-specific manner, orchestrating cardiovascular adaptation to exercise.

The mechanisms by which miRNAs stimulated by exercise mediate cardioprotection are broadly categorized according to their effects on cardiomyocyte growth and survival, mitochondrial function and energy metabolism, anti-inflammatory responses, and endothelial function (Table 1). Collectively, these findings highlight the major role of miRNAs in regulating the cardiovascular effects of exercise and their potential as therapeutic targets for CVD.

**Table 1:** Summary of miRNA studies in exercise-induced cardioprotection.

Category	miRNAs	Role in cardiovascular health	Mechanisms	Clinical implications	References
miRNAs and Cardiovascular Health	miR-126, miR-133a, miR-1, miR-21	Regulate cardiac remodeling, inflammation, oxidative stress, and endothelial function.	- miR-126 promotes angiogenesis via VEGF signaling. - miR-133a inhibits fibrosis and apoptosis.	- Potential biomarkers for cardiovascular disease. - Therapeutic targets for CVD.	(24, 25)
Endothelial function	miR-126, miR-210	Enhance cardiac function, reduce inflammation, and improve endothelial health.	Improvesment of endothelial repair.	- Exercise-induced miRNAs as therapeutic targets. - Biomarkers for CVD progression.	(33, 34)
miRNAs in Cardiomyocyte Growth	miR-1, miR-133a, miR-17-3p	Regulate cardiomyocyte growth and survival, preventing pathological hypertrophy.	- miR-1 targets calmodulin and IGF-1. - miR-133a suppresses RhoA and Cdc42 pathways.	- Potential therapeutic targets for cardiac hypertrophy and heart failure.	(35, 36)
miRNAs in Mitochondrial Function	miR-499, miR-208a	Enhance mitochondrial biogenesis and function, reducing oxidative stress.	- miR-499 regulates mitochondrial fission and fusion proteins. - miR-208a improves energy metabolism.	- Therapeutic targets for mitochondrial dysfunction in CVD.	(37, 38)
miRNAs in Anti-Inflammation	miR-146a, miR-155	Modulate inflammatory responses, reducing chronic inflammation.	- miR-146a targets NF-κB and IRAK1/TRAF6. - MiR-155 regulates macrophage polarization.	- Potential anti-inflammatory therapies for CVD.	(39, 40)

#### 2.2.1 Cardiomyocyte growth and survival

Cardiac hypertrophy is an adaptive response to endurance exercise, characterized by physiological changes such as cardiomyocyte growth, increased mitochondrial density, and enhanced cardiac reserve (41). This type of hypertrophy, or physiological hypertrophy, is delineated from pathological hypertrophy, which entails maladaptive processes such as fibrosis, apoptosis, and reduced contractility, with the frequent progression to heart failure (42). Exercise-mediated miRNAs have important roles as cardiomyocyte survival and growth regulators with a protective role against pathological remodeling and hypertrophy. For instance, miR-1, whose expression is decreased by exercise (43), inhibits major regulators of cardiac hypertrophy, including calmodulin and insulin-like growth factor 1 (IGF-1), to suppress maladaptive growth (35). Similarly, exercise-upregulated miR-133a suppresses pro-hypertrophic signaling pathways like RhoA and Cdc42 to maintain cardiomyocyte homeostasis (36). Some other miRNAs like miR-17-3p are upregulated during exercise-induced hypertrophy, as observed in adult male C57BL/6 mice (8 weeks old) after 4 weeks of treadmill exercise, where miR-17-3p contributed to physiological cardiac growth via modulation of TIMP3 (Tissue inhibitor of metalloproteinases-3) and the phosphoinositide 3-kinase (PI3K)/Akt pathway (44, 45). Additionally, miR-222 has been shown to regulate cardiomyocyte proliferation by targeting HIPK1 (46), while miR-29c and miR-1 play roles in reducing collagen expression and pathological alterations in conditions like obesity-induced hypertrophy. Aerobic exercise training has been demonstrated to restore the levels of these miRNAs, thus preventing pathological cardiac changes and dysfunction (47, 48). Together, these observations underscore the significance of miRNAs in mediating the salutary effects of exercise on cardiac adaptation, and their potential as therapeutic targets for preventing pathological remodeling in CVDs.

#### 2.2.2 Mitochondrial function and energy metabolism

Mitochondrial dysfunction and impaired energy metabolism are central to the pathogenesis of many CVDs (49). miRNAs that are regulated by exercise, specifically miR-499 and miR-208a, have been shown to enhance mitochondrial biogenesis and function by targeting genes involved in oxidative phosphorylation and mitochondrial dynamics (37). For example, miR-499 not only modulates the expression of dynamin-related protein 1 (Drp1), a key regulator of mitochondrial fission, but also represses calcineurin and fission protein 1 (Fis1), thereby promoting mitochondrial fusion over fission. This shift enhances mitochondrial network integrity and bioenergetic efficiency in cardiomyocytes (23). Additionally, miR-499 suppresses the expression of Sox6, a transcriptional repressor of slow-twitch muscle fiber genes and mitochondrial enzymes, thereby favoring oxidative metabolism and endurance capacity (50). In line with this, Fathi et al. found that 14 weeks of endurance training significantly increased miR-499 and decreased Sox6 expression in soleus (slow-twitch) muscle of Wistar rats. Together, these data underscore miR-499/Sox6-driven mitochondrial and fiber-type remodeling as key mechanisms of exercise adaptation (51). miR-208a, predominantly expressed in cardiac muscle, plays a crucial role in mitochondrial homeostasis indirectly through the Thyroid hormone receptor–associated protein 1 (THRAP1) and MED13 (Mediator Complex Subunit 13) signaling axis (38). Moreover, miR-208a can influence metabolic substrate utilization in the heart by suppressing GATA4, a transcription factor involved in mitochondrial gene regulation (52). Collectively, both miR-499 and miR-208a coordinate mitochondrial remodeling and oxidative metabolism in response to physical activity, contributing to the improved metabolic flexibility and resilience of the myocardium during exercise.

#### 2.2.3 Anti-inflammatory effects

Chronic inflammation is a significant contributor to the development of cardiovascular disease (53), and exercise-induced miRNAs have a key function in the regulation of inflammatory processes. For example, miR-146a, which is upregulated by moderate-intensity aerobic exercise in elderly human participants with rheumatoid arthritis (mean age ∼65 years), contributes to anti-inflammatory responses through targeting IL-1 receptor-associated kinase 1 (IRAK1) and TNF receptor-associated factor 6 (TRAF6), key adaptor molecules in the Toll-like receptor (TLR) and Nuclear factor kappa B (NF-κB) signaling pathways By inhibiting these targets, miR-146a reduces the nuclear translocation of NF-κB, leading to decreased transcription of pro-inflammatory cytokines such as tumor necrosis factor alpha (TNF-α), IL-6 (Interleukin 6), and IL-1β. This has been confirmed by observed reductions in serum levels of these cytokines following exercise in miR-146a-upregulated models (39). Similarly, miR-155, another inflammation-associated miRNA, displays context-dependent effects (40). In chronic inflammation settings, such as atherosclerosis, exercise-induced downregulation of miR-155 attenuates macrophage M1 polarization by relieving inhibition of SOCS1 (Suppressor of Cytokine Signaling 1), thereby favoring a shift toward the anti-inflammatory M2 phenotype. This switch is associated with reduced expression of iNOS (inducible nitric oxide synthetase), IL-12, and other pro-inflammatory markers, and increased expression of arginase-1 and IL-10, markers of tissue repair and resolution of inflammation (54, 55). Additionally, miR-21, upregulated by moderate-intensity aerobic training, modulates the PTEN/AKT signaling pathway (56), leading to enhanced activation of eNOS (Endothelial Nitric Oxide Synthase) and suppression of vascular inflammation (57). miR-21 also indirectly inhibits NF-κB activation by targeting PDCD4, a tumor suppressor and pro-inflammatory gene (58), contributing to lower levels of circulating CRP (c-reactive protein) and adhesion molecules such as VCAM-1 (vascular cell adhesion molecule-1) and ICAM-1 (intercellular adhesion molecules**)** in exercise-conditioned individuals (59, 60). Taken together, these miRNAs mediate anti-inflammatory effects of exercise through targeted suppression of upstream regulators in pro-inflammatory cascades and favorable modulation of systemic inflammatory biomarkers. Their consistent changes post-exercise highlights their potential as therapeutic targets for inflammation-driven CVDs.

#### 2.2.4 Endothelial function and angiogenesis

The endothelium is essential for the maintenance of vascular homeostasis (61), and exercise-induced miRNAs have been found to improve endothelial function as well as induce angiogenesis. For instance, exercise-induced upregulated miR-126 targets negative regulators of the VEGF pathway, such as sprouty-related EVH1 domain-containing protein 1 (SPRED1) and phosphoinositide-3-kinase regulatory subunit 2 (PIK3R2), to promote endothelial cell proliferation and angiogenesis (33). Similarly, miR-210, another exercise-responsive miRNA, was found to promote endothelial cell survival under hypoxic conditions *in vitro*, using human umbilical vein endothelial cells (HUVECs), where it inhibited apoptosis and mitochondrial dysfunction through targeting PDK1 (3-Phosphoinositide-dependent protein kinase-1) (34). These miRNAs, collectively, increase blood perfusion and vascular health, thereby reducing the risk of ischemic complications and other vascular diseases (62, 63).

## 3 Long non-coding RNAs in exercise-mediated cardioprotection

### 3.1 Overview of lncRNAs

lncRNAs are a class of non-coding RNA molecules longer than 200 nucleotides that regulate gene expression at the transcriptional, post-transcriptional, and epigenetic levels. Unlike miRNAs, which primarily suppress gene expression, lncRNAs can act as enhancers, repressors, or scaffolds, modulating multiple signaling pathways (64). In the cardiovascular system, lncRNAs play significant roles in cardiac development, fibrosis, hypertrophy, and vascular function. The dysregulation of some lncRNAs has been involved in various CVDs such as heart failure, atherosclerosis, and MI. Increasing evidence suggests that exercise regulates the expression of lncRNAs, and this is involved in its cardioprotective effect through influencing gene regulatory networks in cardiac remodeling, inflammation, and metabolism (65).

### 3.2 Exercise-induced lncRNA expression changes

Physical activity has emerged as a potent modulator of lncRNA expression, with significant implications for cardiovascular health (66). Exercise-induced changes in lncRNA expression play key roles in processes such as cardiac hypertrophy, fibrosis, mitochondrial function, and angiogenesis (66). These effects are mediated by complex molecular interactions involving chromatin-modifying complexes, transcription factors, miRNAs, and RNA-binding proteins.

Several lncRNAs have been empirically shown to respond to exercise stimuli in the heart, with evidence from both animal and human models.

Metastasis-Associated Lung Adenocarcinoma Transcript 1 (MALAT1) was found to be downregulated in a rat model of chronic heart failure subjected to 6 weeks of aerobic treadmill training (5 days/week), accompanied by improved cardiac function and suppression of the PI3K/Akt pathway (67). Likewise, the lncRNA Growth Arrest-Specific Transcript 5 (GAS5) was upregulated in Wistar rats after MI following high-intensity aerobic exercise (66, 68). Given that GAS5 is known to regulate the miR-217/SIRT1 pathway a mechanism implicated in both cancer and fibrosis, this provides a plausible molecular link through which exercise may attenuate cardiac remodeling (69). Exercise-induced expression of lncRNA H19 has been demonstrated to contribute to cardioprotection following MI (70). Mechanistically, H19 exerts its protective effects through multiple pathways: it acts as a competing endogenous RNA for miR-675-5p, thereby promoting angiogenesis, inhibiting cardiomyocyte apoptosis, and improving cardiac function (71). Additionally, H19 directly binds to miR-103/107, suppressing their activity and enhancing the expression of Fas-associated protein with death domain (FADD), which helps prevent oxidative stress-induced cardiomyocyte necrosis (72). It also alleviates myocardial injury via miR-139/Sox8 signaling and activates autophagy to reduce infarct size and enhance cardiac performance (73, 74). These findings highlight lncRNA H19 as a key mediator of the beneficial effects of exercise on post-infarction cardiac remodeling. Importantly, Mhrt779 (Myosin heavy chain-associated RNA transcript), a cardiac-specific lncRNA, was significantly upregulated in C57BL/6 mice following 3 weeks of swimming exercise, and its elevated expression persisted one week post-training. This lncRNA interacts with Brg1 (Brahma-related gene 1) to inhibit maladaptive cardiac remodeling, contributing to the “antihypertrophic memory” observed in trained hearts (75). Exercise training also inhibits MIAT, a pro-fibrotic lncRNA that promotes fibrosis by sequestering microRNA-24 (miRNA-24) and activating the Transforming Growth Factor-beta (TGF-β) pathway (76).

In a rat model of MI induced, Farsangi et al. investigated the impact of a moderate-intensity aerobic exercise protocol (50 min/day, 5 days/week for 4 weeks) on the expression of MI-associated long non-coding RNAs. Their findings showed that exercise significantly decreased the elevated expression of the pro-fibrotic lncRNA MIAT, restored the reduced expression of H19 to near-normal levels, and increased the expression of GAS5. These molecular alterations were accompanied by reduced cardiac fibrosis and apoptosis, as well as improved hemodynamic parameters and contractility indices. This study provides direct experimental evidence that aerobic exercise modulates specific lncRNAs involved in post-infarction remodeling in the heart of Wistar rats (68).

**Figure 1:**
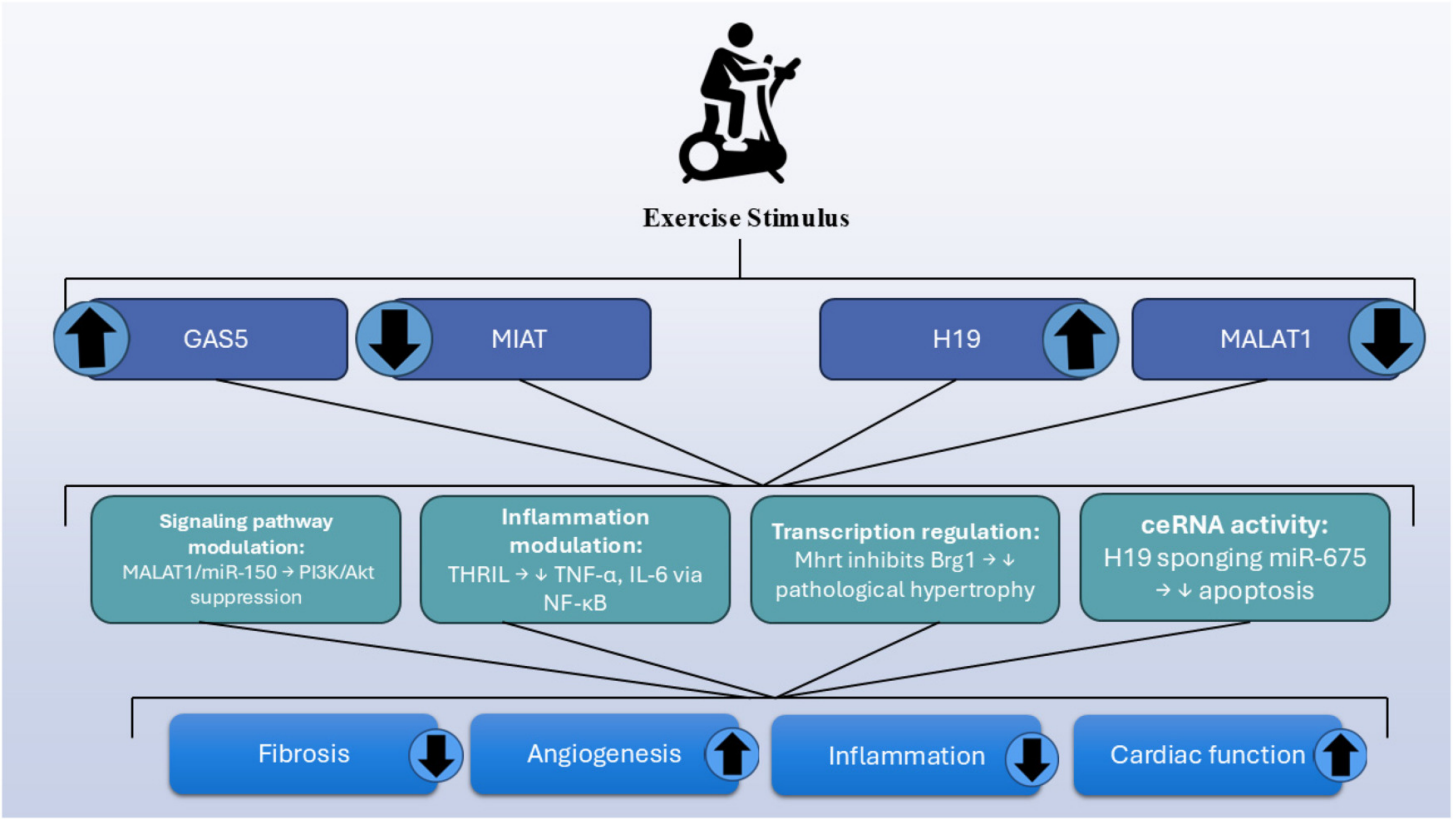
Overview of exercise-regulated lncRNAs and their cardioprotective mechanisms.

These empirical examples underline that lncRNA regulation by exercise is both tissue- and model-dependent, and that specific transcripts exhibit robust expression changes in the heart in response to regular aerobic activity. Such findings strengthen the rationale for targeting exercise-responsive lncRNAs in future cardiovascular therapies (Figure 1). In a chronic heart failure model using adult male Sprague-Dawley rats, six weeks of aerobic treadmill training (5 days/week) improved cardiac function by downregulating the lncRNA MALAT1 and upregulating miR-150-5p, thereby modulating the PI3K/Akt signaling pathway. These findings suggest that aerobic exercise exerts therapeutic effects in chronic heart failure through the MALAT1/miR-150-5p/PI3K/Akt regulator*y* axis (67). These findings emphasize the central function of exercise-induced lncRNA regulation in cardiovascular adaptation and protection against disease and shed new light on the molecular mechanisms of the cardioprotective action of exercise and pave the way for new therapeutic approaches targeting lncRNAs for cardiovascular disease.

Aerobic training of exercise modulates the expression of miRNAs and lncRNAs, which are associated with improved cardiovascular health. Treadmill exercise enhances cardiac function and prevents left ventricular remodeling in heart failure by suppressing MALAT1 and upregulating miRNA-150. Aerobic exercise further reduces cardiomyocyte apoptosis and fibrosis in MI models by restoring the expression of lncRNAs such as H19, MIAT, and GAS5. Exercise also suppresses MIAT, a fibrotic pro-fibrotic lncRNA that promotes fibrosis by sequestering miRNA-24 and activating the TGF-β pathway.

### 3.3 Mechanisms of lncRNA-mediated cardioprotection

LncRNAs exert their cardioprotective effects through a variety of mechanisms, many of which are enhanced or modulated by physical activity. These mechanisms include acting as competing endogenous RNAs (ceRNAs), interacting with chromatin modifiers, regulating transcription factors, and modulating inflammatory responses (Table 2) (77). By integrating these diverse functions, lncRNAs play a pivotal role in mediating the beneficial effects of exercise on heart health, making them promising candidates for novel therapeutic interventions.

**Table 2:** Summary of lncRNA studies in exercise-induced cardioprotection.

Category	lncRNAs	Role in cardiovascular health	Mechanisms	Clinical implications	References
lncRNAs and Cardiovascular Health	H19, MALAT1, MIAT, GAS5	Regulate cardiac hypertrophy, fibrosis, oxidative stress, angiogenesis and enhance mitochondrial function.	- Exercise downregulates MALAT1 and upregulates miR-150. - MIAT inhibition reduces fibrosis. - H19 acts as a ceRNA for miR-675. - MALAT1 regulates PI3K/Akt signaling.	- Potential biomarkers for heart failure. - Therapeutic targets for cardiac remodeling.	(67, 68, 71)
lncRNAs in ceRNA Activity	H19, CHRF	Act as molecular sponges for miRNAs, regulating gene expression.	- H19 sequesters miR-675. - CHRF acts as a ceRNA for miR-489.	- Potential therapeutic targets for cardiac hypertrophy and apoptosis.	(77, 78)
lncRNAs in Transcription Regulation	Mhrt, Mhrt779	Regulate transcription factors involved in cardiac remodeling.	- Mhrt binds to Brg1, inhibiting pro-hypertrophic genes. - Mhrt779 modulates HDAC2/AKT/GSK3β.	- Therapeutic targets for pathological cardiac hypertrophy.	(75)
lncRNAs in Inflammation	THRIL	Modulates inflammatory responses, reducing chronic inflammation.	- THRIL regulates TNFα and IL-6 expression. - Involved in NF-κB signaling.	- Potential anti-inflammatory therapies for CVD.	(79)

#### 3.3.1 ceRNA activity

One of the key mechanisms by which lncRNAs mediate cardioprotection is by acting as ceRNAs or molecular sponges. LncRNAs can bind miRNAs and inhibit their interaction with their target mRNAs, thereby modulating gene expression. For example, the lncRNA H19 has been shown to act as a sponge for miR-675, a miRNA involved in the regulation of cardiomyocyte hypertrophy and apoptosis. By sequestering miR-675, H19 promotes cardiomyocyte survival and reduces pathological remodeling. Similarly, the lncRNA CHRF (Cardiac Hypertrophy-Related Factor) functions as a ceRNA for miR-489, which targets genes involved in cardiac hypertrophy. Exercise-induced upregulation of CHRF has been associated with reduced hypertrophy and improved cardiac function (77, 78).

#### 3.3.2 Regulation of transcription factors

LncRNAs have been shown to play a significant role in the regulation of transcription factors involved in cardiovascular health directly or indirectly. For instance, the lncRNA Mhrt interacts with the chromatin-remodeling factor Brg1, which is involved in pathological hypertrophy. The interaction of Mhrt inhibits Brg1 binding to pro-hypertrophic gene promoters, thereby preventing maladaptive cardiac remodeling (80). Similarly, the lncRNA Mhrt779 has been shown to be upregulated following three weeks of swimming exercise in adult male C57BL/6 mice (8–10 weeks old), with its levels remaining elevated even one week after exercise cessation. This prolonged expression of Mhrt779 was associated with exercise-induced hypertrophic preconditioning and increased resistance to pathological cardiac hypertrophy in murine models. Mhrt779 accomplishes this through interaction with Brg1 and modulating the HDAC2/AKT/GSK3β pathway, reinforcing further the roles of lncRNAs as mediators for exercise-induced heart protective effects (75).

#### 3.3.3 Modulation of inflammatory responses

Among the main causatives of the progress of cardiovascular illnesses is persistent inflammation, and lncRNAs play a fundamental role in the regulation of inflammatory response to physical exercise (7). As an example, the THRIL (TNFα and Heterogeneous Nuclear Ribonucleoprotein L-Related Immunoregulatory LncRNA) lncRNA regulates inflammatory cytokine TNFα and IL-6 gene expression through its interaction with hnRNPL, a protein involved in mRNA splicing and stability (81). Moreover, THRIL has also been demonstrated to be regulated after exercise stress, such as in a half marathon race, reflecting its involvement in exercise-induced inflammation regulation. Aside from its mechanism of action within the NF-κB pathway, THRIL is most probably to influence other pathways involved in the regulation of inflammation as well, further contributing to its role in exercise, inflammation, and cardiovascular health interaction (79). By regulating these pathways, exercise-induced lncRNAs contribute to the reduction of chronic inflammation and its detrimental effects on cardiovascular health.

#### 3.3.4 Therapeutic implications

The discovery of exercise-responsive lncRNAs and their cardioprotective functions has provided new possibilities for the development of lncRNA-based therapies. For instance, synthetic lncRNA mimics or inhibitors may be employed to regulate the expression of particular lncRNAs in patients with CVDs, thus improving cardiac function, decreasing inflammation, and promoting vascular health. In addition, circulating lncRNAs, which are stable in biofluids such as blood, can serve as biomarkers for monitoring the cardiovascular adaptations to exercise and guiding personalized therapeutic strategies. In conclusion, lncRNAs play a central role in mediating the cardioprotective effects of exercise through diverse mechanisms, including ceRNA activity, chromatin remodeling, regulation of transcription factors, and modulation of inflammatory responses. A deeper understanding of these mechanisms may pave the way for innovative therapeutic strategies that harness the power of lncRNAs to prevent and treat CVDs, ultimately improving outcomes for patients worldwide (Table 2).

## 4 Epigenetic modifications and their role in exercise-induced cardioprotection

### 4.1 Overview of epigenetics in cardiovascular health

Epigenetics refers to heritable gene expression changes that occur without alterations in the DNA sequence. These modifications include DNA methylation, histone modifications, and non-coding RNA interactions, all of which play crucial roles in regulating gene activity in response to environmental stimuli, including exercise (82). In the cardiovascular system, epigenetic regulation influences key processes such as cardiac remodeling, inflammation, oxidative stress, and endothelial function. Epigenetic process dysregulation has been implicated in the pathogenesis of cardiovascular disease such as atherosclerosis, heart failure, and hypertension (83). Exercise has been shown to induce beneficial epigenetic modifications that promote cardioprotection, highlighting the potential for targeting epigenetic pathways in cardiovascular disease prevention and treatment (Table 3).

**Table 3:** Summary of epigenetic modifications in exercise-induced cardioprotection.

Category	Examples	Role in cardiovascular health	Mechanisms	Clinical implications	References
DNA Methylation	PGC-1α,	Regulates inflammation, oxidative stress, and metabolic health.	- Exercise-induced hypomethylation of PGC-1α enhances mitochondrial biogenesis.	- Potential biomarkers for metabolic and cardiovascular health. - Therapeutic targets for CVD.	(84)
DNA Methylation in Inflammation	ASC, KLF14	Reduces inflammation and improves vascular health.	- ASC methylation reduces IL-1β production. - KLF14 methylation promotes anti-inflammatory effects.	- Therapeutic targets for inflammatory cardiovascular diseases.	(85–88)
Histone Modifications	HDAC4, HDAC1, HDAC2	Modulates cardiac remodeling, fibrosis, and endothelial function.	- Exercise reduces HDAC activity, improving cardiac function. - HDAC4 regulates calcium handling.	- Xercise-induced histone modifications as therapeutic targets. - Biomarkers for heart failure.	(89, 90)
Histone Deacetylation	HDAC4, HDAC1, HDAC2	Reduces fibrosis and improves cardiac function.	- Exercise-induced HDAC4 fragments protect against heart failure. - HDAC1/HDAC2 reverse diabetic heart pathology.	- Therapeutic targets for heart failure and diabetic cardiomyopathy.	(89, 90)

### 4.2 Exercise and DNA methylation

**Figure 2:**
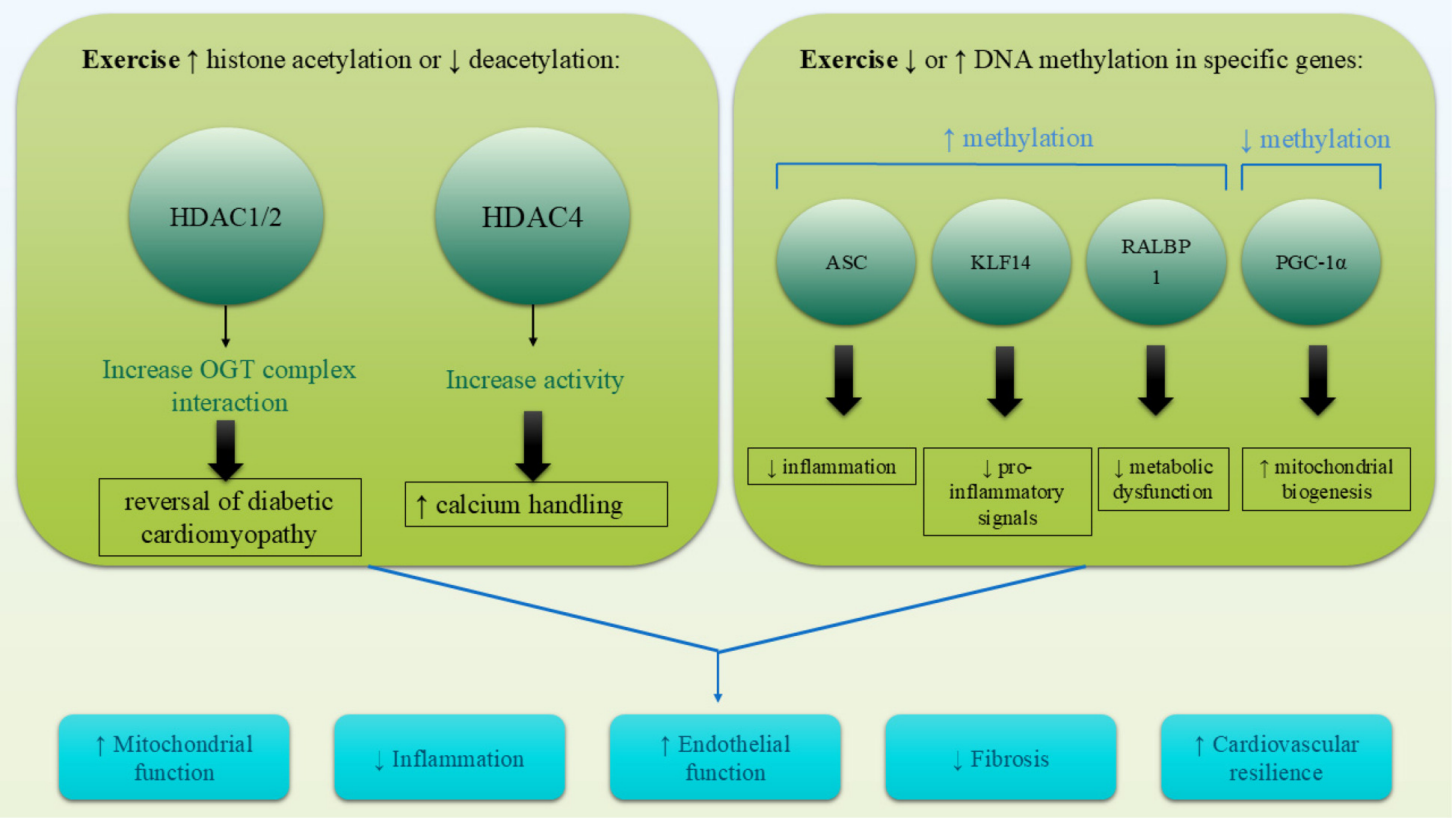
Exercise induces cardioprotective epigenetic modifications through alterations in DNA methylation and histone modifications. Hypomethylation of genes like PGC-1α enhances mitochondrial biogenesis, while hypermethylation of ASC and KLF14 reduces inflammation. Simultaneously, exercise promotes histone acetylation (e.g., PGC-1α, VEGF) and reduces HDAC activity (e.g., HDAC4, HDAC1/2), leading to improved gene expression profiles involved in cardiac repair, energy metabolism, and vascular health.

DNA methylation, which involves the addition of a methyl group to cytosine residues in CpG islands, is a critical epigenetic process to regulate gene expression. Aberrant DNA methylation patterns have been associated with CVDs, contributing to inflammation, vascular dysfunction, and metabolic dysregulation (83). Regular physical activity has been shown to modify DNA methylation patterns in genes related to cardiovascular health, leading to favorable gene expression changes (91). While exercise-regulated DNA methylation in cardiac tissue has not yet been fully documented, the evidence shows that exercise-induced DNA methylation in tissues other than cardiac tissue, such as skeletal muscle and adipose tissue, plays a major role in providing anti-inflammatory effects and metabolic remodeling (92). Such epigenetic changes lead to the decreased prevalence of cardiac disease, which is indicative of the possible systemic impact of exercise on cardiovascular health. Exercise-induced DNA methylation has been recognized as a primary mechanism in the reduction of inflammation, improvement of metabolic health, and lowering of cardiovascular disease risk. Research indicates that exercise increases the methylation of ASC (apoptosis-associated speck-like protein), a gene involved in inflammasome activation, leading to reduced interleukin-1β production and improved outcomes in heart failure (85, 86). Exercise also promotes hypermethylation of Kruppel-like factor 14 (KLF14) and src homology 2 domain-containing transforming protein C1, which are associated with anti-inflammatory effects and healthy vascular aging, respectively (87, 88). Exercise induces hypomethylation of PGC-1α in skeletal muscle, a central controller of mitochondrial biogenesis and energy metabolism (84), while also modulating the methylation of PDK4 and PPARD, genes essential for glucose and fatty acid metabolism (92). Similarly, exercise regulates the methylation of NRF1 and NR4A1, transcription factors linked to insulin sensitivity and mitochondrial function (93). In adipose tissue, exercise upregulates methylation of RALBP1, a gene implicated in metabolic syndrome (94). Collectively, these findings nominate exercise-evoked DNA methylation as a vital element for improving cardiometabolic health and an emerging therapeutic application for prevention against cardiac and metabolic diseases (Figure 2).

### 4.3 Exercise and histone modifications

Histone modifications, including acetylation, methylation, and phosphorylation, influence chromatin structure and gene transcription. Histone acetylation, mediated by histone acetyltransferases (HATs), generally promotes gene expression, while histone deacetylation, facilitated by histone deacetylases (HDACs), leads to gene repression (95). Exercise has been shown to modulate histone modifications in a manner that supports cardiovascular health, such as increasing histone acetylation in genes involved in angiogenesis and mitochondrial biogenesis (96). Additionally, reductions in HDAC activity caused by exercise have been linked to improved cardiac function, reduced fibrosis, and enhanced endothelial homeostasis. For example, exercise-induced fragments of HDAC4 avert heart failure through the regulation of the hexosamine biosynthetic pathway, protein O-GlcNAcylation, and calcium handling (89). Additionally, HDAC1 and HDAC2 contribute to exercise-induced cardioprotection, with evidence showing that exercise promotes the interaction between the mSin3A/HDAC1/HDAC2 complex and O-GlcNAc transferase (OGT), reversing diabetic heart pathology (90). These findings suggest that exercise-induced histone modifications are cardioprotective in heart failure, diabetic cardiomyopathy, and obesity-related cardiac dysfunction. Further studies are needed to elucidate the exact mechanisms of HDACs and investigate other histone modifications, such as methylation, phosphorylation, and ubiquitination, of exercise-induced cardioprotection. Through the regulation of histone modifications, physical training creates a more favorable epigenetic environment conducive to long-term cardiovascular protection.

### 4.4 Epigenetic memory: persistence or reversibility of exercise-induced marks

A critical question in cardioepigenetics is whether exercise-induced epigenetic changes are transient or capable of persisting after cessation of physical activity. Accumulating evidence suggests that while some epigenetic modifications are short-lived and require continued exercise stimuli, others can endure for extended periods, creating what is now referred to as epigenetic memory.

One of the most well-characterized examples is DNA hypomethylation of the PGC-1α promoter, a master regulator of mitochondrial biogenesis. In both human and rodent models, this epigenetic mark has been shown to remain at least partially reduced for several days to weeks following exercise cessation, supporting prolonged oxidative adaptations even during inactivity (97). In a landmark human study using muscle biopsies before and after a 7-week resistance training program, followed by a detraining period and then retraining, researchers identified over 18,000 CpG sites that remained hypomethylated even after inactivity. Notably, genes such as AXIN1, GRIK2, CAMK4, and TRAF1 retained their epigenetic changes, suggesting that prior training leaves a molecular imprint that may facilitate future adaptations (98). On the other hand, histone modifications such as phosphorylation (e.g., H3S10ph) and acetylation (e.g., H3K9ac), while critical for acute transcriptional responses to exercise, tend to be more labile and transient, often reverting to baseline shortly after exercise cessation (99). However, the persistence of these modifications appears to be tissue-specific, dose-dependent, and individualized, influenced by factors such as exercise intensity, age, sex, and genetic background. Some modifications regress quickly upon inactivity, highlighting the importance of exercise regularity in maintaining beneficial epigenetic states (100).

Collectively, these findings suggest that exercise can induce both reversible and persistent epigenetic reprogramming, with DNA methylation-based changes playing a pivotal role in long-term cardiovascular adaptations. This has important implications for the design of personalized exercise programs and the development of “epigenetic dosing” models aimed at maximizing health benefits through sustained molecular signaling.

## 5 Exercise modality, intensity, and their impact on cardiovascular miRNA regulation

Both aerobic and resistance exercise have been shown to modulate the expression of cardiovascular-related miRNAs, but their regulatory effects differ in terms of mechanism, target pathways, and outcomes. Aerobic training, especially when performed at moderate to high intensities for sustained durations (e.g., ≥30 min per session, ≥3 times per week), commonly upregulates miRNAs associated with endothelial function, angiogenesis, mitochondrial biogenesis, and cardioprotection such as miR-126, miR-133a, and miR-210. These effects are often mediated through improved oxidative metabolism and hemodynamic stress adaptation (101). Studies have demonstrated that regular aerobic and high-intensity exercise can increase levels of miR-126 in both the bloodstream and specific tissues, promoting angiogenesis and endothelial function (24).

In contrast, resistance training (RT), typically involving high-intensity, short-duration bouts of mechanical loading, more prominently influences miRNAs related to muscle remodeling, inflammation, and cellular survival (102). For instance, RT has been associated with increased expression of miR-21, miR-222, miR-208b, and miR-499, which are involved in pathways regulating cardiac contractility, fibrosis inhibition, and mitochondrial function (103–105). Moreover, preliminary evidence suggests that the intensity and volume of resistance training may influence the profile of miRNA expression, although direct comparative studies between different loading schemes remain limited (106). Furthermore, combined aerobic and resistance exercise may induce adaptive changes in circulating miRNAs that reflect both metabolic and structural cardiovascular remodeling. For instance, in patients with HFrEF undergoing 15 weeks of combined training, specific plasma miRNAs (e.g., miR-146a, miR-23a) were modulated and associated with VO₂peak improvements and cardiac remodeling markers (107).

Altogether, these findings emphasize that the type, intensity, and duration of exercise are key modulators of miRNA and lncRNA expression patterns, and support the concept of personalized exercise prescriptions to maximize cardiovascular epigenetic benefits.

## 6 Interplay between miRNAs, lncRNAs, and epigenetics

Exercise-induced cardioprotection is coordinated by a highly interactive network of miRNA-lncRNA-epigenetic alteration interactions but not by distinct molecular processes. These factors co-operate to modulate gene expression, govern cell signaling pathways, and trigger adaptive responses in the cardiovascular system (21). Through the creation of a dynamic feedback loop, they increase cardiac resilience, reduce inflammation, and improve vascular function. For instance, lncRNAs can act as molecular sponges for miRNAs, sequestering them and preventing their interaction with target mRNAs. This interaction fine-tunes the regulatory effects of miRNAs on gene expression (108). Conversely, epigenetic changes can also impact the expression of both miRNAs and lncRNAs, creating a bidirectional network of regulation. For instance, DNA methylation and histone acetylation can control miRNA expression such as miR-1 and miR-133, which influence gene regulation regarding cardiac remodeling and function (108). The combined effects of miRNAs, lncRNAs, and epigenetic modifications enhance cardiac resilience by promoting adaptive hypertrophy, reducing fibrosis, and improving mitochondrial function. These molecular changes also attenuate chronic inflammation and oxidative stress, which are key drivers of cardiovascular disease (17). For example, exercise-induced upregulation of miR-126 and lncRNA H19 promotes angiogenesis and endothelial repair, while epigenetic modifications such as histone acetylation of antioxidant genes enhance cellular resistance to oxidative damage (68, 109). Together, these mechanisms create a protective molecular environment that supports long-term cardiovascular health.

## 7 Future directions and research gaps

The discovery of exercise-induced miRNAs and their cardioprotective mechanisms has opened new avenues for therapeutic strategies aimed at mimicking the molecular benefits of physical activity. Synthetic miRNA mimics (e.g., miR-126, miR-133a, miR-146a) and inhibitors (e.g., anti-miR-1 or anti-miR-21) hold potential for modulating key pathways involved in inflammation, fibrosis, apoptosis, and mitochondrial function (110). However, despite encouraging preclinical data, the clinical translation of miRNA-based therapies remains highly challenging. Most findings are derived from animal models, and only a limited number of human trials have progressed to advanced phases. Key limitations include the lack of safe, efficient, and tissue-specific delivery systems, as well as the risk of off-target effects and immune activation due to non-physiological delivery methods. Moreover, the pleiotropic nature of miRNAs regulating multiple mRNA targets raises concerns about unintended downstream consequences (111, 112).

Among the few miRNA-based interventions tested in humans, miR-132-3p inhibition has shown promising translational potential. A first-in-human phase Ib study in heart failure patients demonstrated favorable tolerability and preliminary efficacy, following robust preclinical data in rodent and porcine models (113, 114). Among the promising therapeutic targets, miR-155 inhibition shows strong anti-inflammatory effects in preclinical cardiac inflammation models by modulating macrophage activity and NF-κB signaling. The antimiR-155 drug cobomarsen reached phase I clinical trials for lymphoma but was discontinued in phase II for strategic reasons ( (115, 116). miR-92a-3p is highly expressed in endothelial cells and plays a critical role in vascular and myocardial injury. AntimiR-92a (MRG-110) has demonstrated pro-angiogenic and tissue repair effects in preclinical models, including mice and pigs. Early clinical trials in healthy volunteers have assessed its safety and pharmacodynamics via intravenous and intradermal administration, marking important steps toward therapeutic application (116, 117).

Long-term safety, pharmacokinetics, and chronic administration effects remain largely unexplored, particularly with respect to their interaction with endogenous regulatory networks. Considering these limitations, future research must focus on several critical priorities: conducting well-controlled, longitudinal human studies to validate animal-based findings; improving delivery technologies (e.g., nanoparticle- or exosome-based carriers); and deepening our mechanistic understanding of miRNA–lncRNA–epigenetic interplay. Furthermore, standardizing exercise intervention protocols and characterizing the influence of variables such as age, sex, comorbidities, and exercise dose on molecular responses are essential steps toward clinical applicability.

### 7.1 Clinical applications of exercise-induced epigenetic discoveries

Recent advances in exercise epigenetics have opened promising avenues for clinical translation, particularly in the realm of precision cardiovascular medicine. Epigenetic alterations such as changes in DNA methylation, histone modifications, and non-coding RNA expression represent dynamic, modifiable markers that can serve as both diagnostic tools and therapeutic targets (118). For instance, specific exercise-regulated miRNAs, such as miR-126 and miR-146a, as well as lncRNAs like H19 and GAS5, have shown promise as circulating biomarkers of cardiovascular stress and vascular inflammation. Their stability in blood and detectability by qPCR make them suitable for liquid biopsy approaches to assess cardiovascular risk or training response (119). In parallel, the development of epigenetic drugs targeting enzymes like DNA methyltransferases (DNMTs), histone deacetylases (HDACs), or RNA-based therapeutics (e.g., miRNA mimics/inhibitors) offers potential for mimicking or enhancing the beneficial molecular effects of exercise particularly in individuals unable to engage in regular physical activity due to advanced disease, disability, or aging. Although these strategies remain largely experimental, their integration into clinical protocols may complement conventional rehabilitation or preventive measures (120).

Integration of individualized epigenetic profiling into clinical practice may enable personalized exercise prescriptions. By assessing baseline epigenetic signatures such as DNA methylation patterns in genes regulating inflammation, mitochondrial function, and vascular homeostasis clinicians could tailor the type, intensity, and duration of exercise to achieve maximal benefit (121, 122). As technologies such as next-generation sequencing and machine learning evolve, the prospect of epigenetically guided exercise interventions is becoming increasingly feasible (123, 124).

These clinical applications position epigenetic discoveries not only as mechanistic insights but also as transformative tools for individualized prevention and treatment of cardiovascular disease.

### 7.2 Expanded future perspectives: standardization, biomarkers, and environmental interactions

To advance the clinical utility of exercise epigenetics, future research must prioritize the standardization of exercise protocols in both animal and human models. Currently, the heterogeneity in exercise modalities (e.g., aerobic, resistance, HIIT), intensities (e.g., moderate vs. vigorous), durations, and frequencies limits the comparability of studies and hinders meta-analysis. whereas aerobic exercise has been shown to increase cardiac expression of lncRNAs like MALAT1 and Mhrt, associated with cardiomyocyte survival (67), resistance-type protocols such as swimming in rodents have been reported to upregulate the cardiac lncRNA Mhrt779, mediating physiological antihypertrophic remodeling via the Brg1/Hdac2/p-Akt/p-GSK3β axis (75, 125).

Similarly, cardiac miRNAs such as miR-126 (angiogenesis) and miR-222 (cell cycle and cardiomyocyte growth) are differentially regulated depending on training load and modality: in diabetic rats, interval training elicited significantly greater myocardial expression of miR-126 and miR-222 compared to continuous exercise (126); in humans, both serial and integrated concurrent exercise sessions induced acute increases in circulating miR-126 and miR-222, underscoring their sensitivity to exercise stimulus ( (127).

Standardized protocols will thus be essential to define molecular thresholds for cardioprotective effects, especially in relation to non-coding RNA regulation such as miRNAs and lncRNAs. Moreover, epigenetic modifications particularly DNA methylation of promoters such as PGC-1α, a key regulator of mitochondrial biogenesis are emerging as critical mediators of exercise-induced cardiovascular adaptation and warrant standardized investigation (128).

Another emerging area involves exploring the modulatory effects of lifestyle and environmental factors, including diet, sleep quality, chronic stress, circadian disruption, and pollution exposure, on the cardiac epigenome (129, 130). These variables can enhance or suppress the molecular benefits of exercise by interfering with chromatin remodeling or non-coding RNA expression. For instance, sleep deprivation alters circulating miRNA profiles, indicating its potential to blunt ncRNA-mediated exercise responses (131–133), while antioxidant-rich diets and polyphenols have been shown to modulate lncRNA expression in cardiovascular contexts (134). Integrative multi-omic profiling and systems biology approaches will be essential to dissect these interactions and move toward personalized exercise interventions.

In summary, by addressing these key priorities protocol standardization, biomarker discovery, and environmental interaction mapping future research can bridge the gap between experimental findings and clinical translation, ultimately contributing to precision cardiovascular medicine grounded in epigenetic regulation.

### 7.3 Technical and bioethical challenges for lncRNA-based therapies

Despite the growing evidence supporting the role of lncRNAs in cardiovascular adaptation to exercise, the clinical translation of lncRNA-based therapies remains particularly challenging. Delivery challenges are especially pronounced due to the structural complexity and diverse modes of action of lncRNAs, which often interact with chromatin, RNA, or proteins in a context-specific manner (135). Additionally, the low sequence conservation of lncRNAs across species complicates the extrapolation of preclinical findings to human physiology (65).

From a bioethical standpoint, manipulating lncRNAs raises concerns about unpredictable off-target effects, long-term epigenetic alterations, and the persistence of synthetic molecules in human tissues (136). These risks are particularly relevant in otherwise healthy individuals where preventive interventions may be considered. Careful ethical scrutiny and regulatory oversight are therefore essential, especially when genome-editing tools or chronic systemic delivery methods are involved (66).

Looking ahead, non-pharmacological approaches that harness endogenous regulatory mechanisms—such as personalized exercise prescriptions informed by individual lncRNA expression profiles—may offer safer and more physiologically harmonious alternatives (137). Emerging liquid biopsy platforms may allow clinicians to monitor circulating lncRNAs in real time, enabling dynamic optimization of training intensity, duration, and modality (138). Furthermore, integrating multi-omics profiling with machine learning could help identify patient subgroups most likely to benefit from lncRNA-targeted strategies, paving the way for precision exercise-based cardiovascular medicine (139).

## 8 Conclusions

The coordinated interaction between miRNAs, lncRNAs, and epigenetic modulation in exercise-induced cardioprotection is an exciting area with extensive therapeutic potential. By elucidating the complex crosstalk of these molecular regulators, researchers can develop new interventions for cardiovascular disease prevention and therapy. Future studies must focus on bridging existing knowledge gaps and translating preclinical findings into the clinic, ultimately improving cardiovascular outcomes for patients worldwide.
